# Irreversible Electroporation in Liver Cancers and Whole Organ Engineering

**DOI:** 10.3390/jcm8010022

**Published:** 2018-12-25

**Authors:** Aman Saini, Ilana Breen, Sadeer Alzubaidi, Yash Pershad, Rahul Sheth, Sailendra Naidu, M. Grace Knuttinen, Hassan Albadawi, Rahmi Oklu

**Affiliations:** 1 Department of Vascular and Interventional Radiology, Laboratory for Minimally Invasive Therapeutics, Mayo Clinic, Phoenix, AZ 85054, USA; Saini.Aman@mayo.edu (A.S.); Breen.Ilana@mayo.edu (I.B.); alzubaidi.sadeer@mayo.edu (S.A.); ypershad@stanford.edu (Y.P.); Naidu.Sailen@mayo.edu (S.N.); knuttinen.grace@mayo.edu (M.G.K.); albadawi.hassan@mayo.edu (H.A.); 2 Department of Interventional Radiology, MD Anderson Cancer Center, Houston, TX 77030, USA; RASheth@mdanderson.org

**Keywords:** irreversible electroporation, liver cancer, HCC, whole-organ engineering

## Abstract

Liver cancers contribute significantly to cancer-related mortality worldwide and liver transplants remain the cornerstone of curative treatment for select, early-stage patients. Unfortunately, because of a mismatch between demand and supply of donor organs, liver cancer patients must often wait extended periods of time prior to transplant. A variety of local therapies including surgical resection, transarterial chemoembolization, and thermal ablative methods exist in order to bridge to transplant. In recent years, a number of studies have examined the role of irreversible electroporation (IRE) as a non-thermal local ablative method for liver tumors, particularly for those adjacent to critical structures such as the vasculature, gall bladder, or bile duct. In addition to proving its utility as a local treatment modality, IRE has also demonstrated promise as a technique for donor organ decellularization in the context of whole-organ engineering. Through complete non-thermal removal of living cells, IRE allows for the creation of an acellular extra cellular matrix (ECM) scaffold that could theoretically be recellularized and implanted into a living host. Here, we comprehensively review studies investigating IRE, its role in liver cancer treatment, and its utility in whole organ engineering.

## 1. Introduction

Liver cancers including hepatocellular carcinoma (HCC) and intrahepatic cholangiocarcinoma (ICC) contribute significantly to worldwide cancer mortality. In the United States in 2017, there were an estimated 40,710 new primary liver cancer cases [1]. Furthermore, from the period of 2000–2016, liver cancer death rates for adults aged 25 and over increased by 43% [2]. Although surveillance of high-risk patients and advances in treatment regimens are prolonging survival, long-term outcomes remain poor. For patients with early-stage disease, surgical resection and local therapies play an integral role in the treatment paradigm, while offering a bridge to liver transplant, which is considered curative [3,4,5,6,7,8,9]. However, increased nationwide demand and decreased supply of donor livers make organ procurement difficult, necessitating more effective therapies and alternate organ sources. In recent years, irreversible electroporation (IRE) has emerged as a solution to fulfill these needs and may play a central role in the future treatment of patients with liver cancers. 

### Electroporation: Technical Description, Procedure, and Role in Medicine

**Figure 1:**
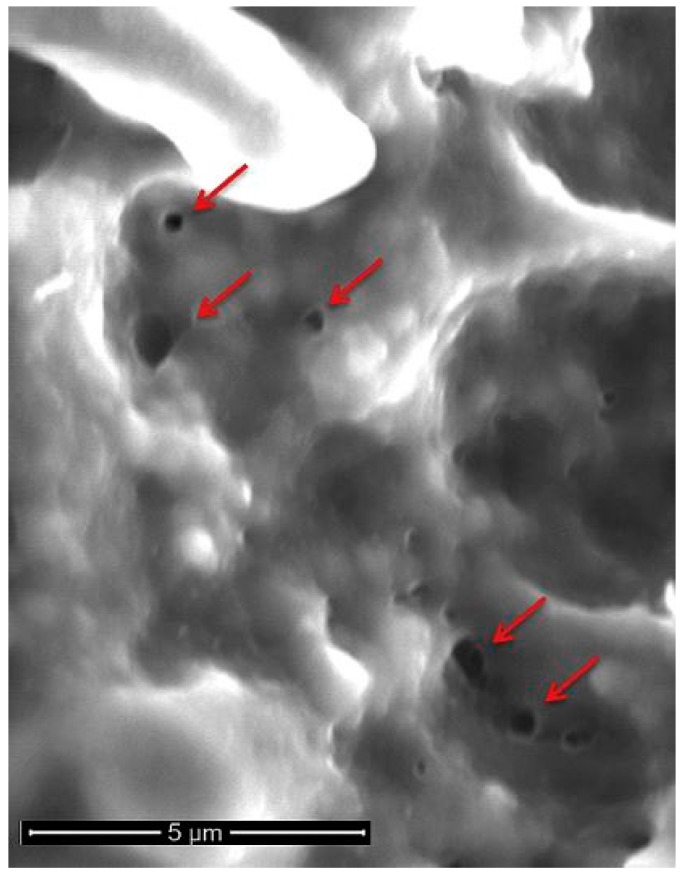
Scanning electron microscopy of irreversible electroporation (IRE)-ablated porcine liver. Nano-sized pores (red arrows) are visible on the cell membrane. Reproduced with permission from Jourabchi, N. et al., Gastrointestinal Interventions; published by Elsevier, B.V., 2014.

Electroporation is a well-known laboratory technique that has evolved with time and has widespread applications. It is a reversible biophysical process that uses short, high-voltage electric pulses to create an external electric field that exceeds cell membrane capacitance, creating nanoscale pores in the lipid bilayer of cell membranes. The electric pulses are often delivered via plate or needle electrodes connected to a computer controlled pulse generator. The threshold potential required to induce membrane pores has been reported to be between 200 millivolts and 1 volt, with pulse durations in the order of milliseconds to nanoseconds [10]. Pore formation occurs within microseconds, while membrane resealing happens in the order of minutes [10]. At higher applied field strengths in the kilovolt range, the pore formation process becomes irreversible, leading to changes in osmotic gradients, cellular homeostasis, and eventual cell death (Figure 1) [11]. 

When used as a tumor ablation method, IRE most often involves the placement of a single bipolar or multiple monopolar radiopaque 16 or 18 gauge probes that are 15 or 25 cm in length, arranged peritumorally with sufficient normal parenchymal margins, and placed at pre-determined distances (typically 1.5–2 cm) from each other [12]. Although treatment parameters vary on a per case basis, field strengths between 1000 and 3000 volts and pulse durations lasting 20–100 microseconds have been used clinically [12,13,14]. The number and pattern of pulses may also vary and pulses are electrocardiographically gated to avoid arrhythmias. Patients are often placed under general anesthesia with muscle paralysis to prevent movement during pulse administration. IRE-mediated damage is non-thermal and specific to cell membranes, sparing other critical structures like vessels, nerves, bile ducts, and urethra. The non-thermal nature of this ablation technique distinguishes it from other commonly used thermal methods like microwave and radiofrequency ablation techniques. The exact mechanism of cell death in IRE is under debate and apoptosis, necroptosis, and pyroptosis have been observed in the laboratory [15,16,17]. 

The role of IRE in medicine has predominantly been studied within the context of interventional oncology, and many studies have examined the safety, efficacy, and clinical outcomes for a variety of cancers treated with IRE [18,19,20,21]. Pre-clinical studies have also examined the role of IRE as a tool in gene therapy [22], in vascular medicine, and in the decellularization of donor tissues in the context of whole-organ tissue engineering. This promising and exciting approach to regenerative medicine relies on using IRE to decellularize an entire donor organ while maintaining its native extracellular matrix (ECM) and three-dimensional architecture. The remaining acellular scaffold could then theoretically be reseeded with cells prior to being used as a donor organ. In this review, we examine the emerging role of IRE in the treatment of various hepatic malignancies with a focus on the most relevant clinical studies to date. The utility of IRE as an organ decellularization method in the context of whole-organ tissue engineering is also reviewed. Finally, we briefly review techniques for recellularization of acellular organ scaffolds and potential sources of cells. 

## 2. Irreversible Electroporation in Hepatic Malignancies

**Figure 2:**
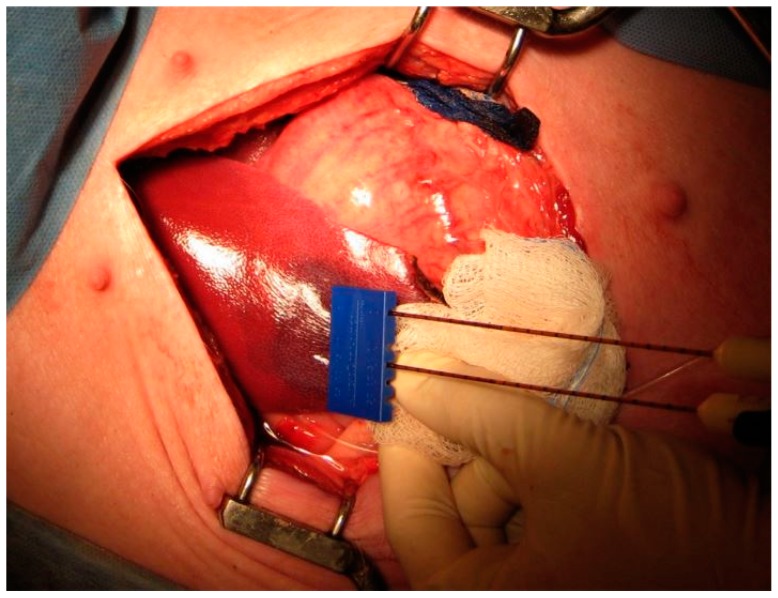
IRE ablation in a porcine liver. Dual IRE probes are spaced 1.5 cm apart on the surface of a pig liver. The dark red area adjacent to the probe tip indicates IRE-ablated liver tissue. Reproduced with permission from Au, J. et al., Surgery; published by Elsevier Inc., 2013.

Surgical resection remains an effective treatment for liver tumors; however, a very small percentage of patients are considered resectable at diagnosis as a result of advanced disease or poor baseline liver function. For these patients who may require local therapy, various thermal ablative methods including microwave ablation, radiofrequency ablation (RFA), and cryoablation can be used. However, the effectiveness and utility of these modalities is limited by tumor size, proximity to adjacent structures including organs and blood vessels, and the heat or cool sink effects that result from temperature reductions (or increases in the case of cryoablation) due to perfusion of adjacent large blood vessels. IRE ablation is able to overcome these issues, particularly when used on tumors adjacent to vital structures, as it does not rely on thermal energy to induce cell death, and the ablative zone can be defined by probe placement [6]. The end result is a region of cell death that does not typically exhibit gradual changes in tissue damage, as seen in thermal ablation [11] (Figure 2). 

### 2.1. Safety and Early Efficacy

A number of clinical studies have evaluated the safety and efficacy of IRE ablation for liver tumors with promising initial results. In a 2013 study of 44 patients undergoing surgical or percutaneous IRE for hepatic tumors (HCC, liver metastases) adjacent to sensitive structures (gall bladder, portal and hepatic veins, and bile ducts), Cannon et al. noted a total of nine adverse events in five patients, the worst of which included biliary stent occlusion, cholangitis, neurogenic bladder, and flank pain [23]. All complications were resolved in 30 days, and no treatment related deaths were noted. Furthermore, the investigators confirmed the importance of electrocardiogram (ECG) synchronization as a means of avoiding arrhythmias during IRE treatment, a complication that had been noted in previous studies. With respect to treatment efficacy, 100% of the tumors demonstrated complete ablation (a surrogate for local therapy efficacy) with no evidence of residual tumor at 3 months on follow up imaging. However, at 6 months, local recurrence free survival (LRFS) decreased to 94.6% and at 12 months, it decreased to 59.5%. As this study did not present recurrence outcomes by tumor origin, presumably because of the small sample size, it is difficult to assess whether this one year recurrence rate is due to treatment failure or the aggressiveness of the primary tumor type. The investigators did note, however, a relationship between tumor size and recurrence, with tumors over 4 cm trending towards higher rates of recurrence. A similar study assessing IRE safety was conducted by Cheung and colleagues on 11 patients with 18 unresectable HCC lesions in close proximity to critical structures. In this small patient group, no serious complications were noted, particularly those involving adjacent organs including the gallbladder, colon, or heart. Furthermore, cardiac arrhythmias did not occur in any patients. The efficacy of IRE ablation in these patients, however, was strongly dependent on tumor size with a complete response observed in 93% of patients with tumors <3 cm, while all patients with tumors >3 cm had residual disease, even after a second IRE treatment [24]. In a larger prospective multi-institution study with 150 consecutive patients treated with IRE, 39.5% of whom had liver cancers, Phillips et al. similarly observed low rates of complications with an overall attributable complication rate of 13.3% and an attributable major complication rate of 4.4% [12]. This low complication rate persisted for patients with larger or more numerous lesions, even for lesions with increased vascular invasion. With respect to treatment efficacy, 10.1% of tumors demonstrated incomplete ablation after the first procedure. However, unlike the results from Cheung et al., there were no significant differences in ablation efficacy with respect to tumor size, although the median size of tumors in the patient subgroup with the largest tumors was only 3.9 cm. Collectively, the results of these studies, in addition to numerous others involving the safety and efficacy of IRE ablation in liver tumors [14,25,26], suggest that IRE is a safe local ablative method with a relatively low major complication rate. Further studies in larger patient populations are warranted to determine the ideal patient population to be treated with this technique. 

### 2.2. Recurrence and Survival Outcomes for IRE-Ablated Liver Tumors

To date, there has been a limited number of studies examining the mid- to long-term tumor control and survival outcomes of patients with IRE ablated liver tumors. In a single center non-randomized trial of 30 patients with 38 malignant hepatic lesions (23 metastatic colorectal cancer lesions, 8 HCC lesions, and 7 other metastases) and a median tumor size of 2.4 cm, Fruhling and colleagues demonstrated local recurrence rates of 21.1% and 34.2% at 3 and 6 months, respectively, for both HCC and colon cancer [27]. There was no significant difference in terms of local recurrence between colon cancer and HCC patients, although the number of patients in this study, particularly in the HCC group, limit statistical power. This study had other limitations including the inclusion of six patients with tumors larger than 3 cm, which was contrary to the original inclusion criteria. These patients were palliatively treated patients whose inclusion may have affected outcomes. In another investigation by Niessen et al., the long term recurrence outcomes for 71 primary (*n* = 35) and secondary (*n* = 36) inoperable liver cancer patients undergoing IRE ablation were assessed [28]. With a median tumor size of 2.3 cm and a follow up time of three years, 31% of tumors demonstrated a local recurrence and the overall median survival was 26.3 months. Furthermore, the median survival for primary versus secondary liver cancers was 26.8 and 19.9 months, respectively, although this difference was not significant. Additionally, tumors >3 cm or patients with three or more lesions demonstrated significantly lower overall survival rates. These results mirror those of previously discussed studies and highlight the link between small tumor size and improved IRE outcomes. Additionally, the non-significant difference in survival times between primary and secondary liver tumors, which is most likely the result of underlying tumor biology and aggressiveness, needs to be further explored to determine the tumor type most suitable for IRE ablation. 

More recently, Langan et al., in a series of 40 patients with 77 hepatic lesions undergoing open IRE with a median follow-up time of 26 months, demonstrated a cumulative incidence of local recurrence of 13.4% [29]. Furthermore, no local recurrences were noted in 19 months after IRE treatment. Factors that were associated with increased local recurrence were increasing size of the ablation zone and increased body mass index. Overall, this study had several interesting outcomes that suggest the promising potential of IRE. First, the low incidence of local recurrence and no local recurrences after 19 months suggest durable control in this specific patient population. Second, in 13% of patients who underwent the first portion of a two-stage hepatectomy and then received IRE ablation within the liver remnant, IRE ablation was found to downstage all patients, allowing for the second portion of the hepatectomy to proceed [29]. This outcome highlights the potential of IRE to further expand the pool of candidates for liver tumor resection. Finally, the investigators speculated that the link between increasing body mass index (BMI) and increased local recurrence could possibly be the result of fatty infiltration of the liver, which may affect electrical conductivity and thus IRE ablation efficacy overall. This finding warrants further investigation and could potentially help to further guide patient selection. When considered collectively, these studies investigating recurrence and survival outcomes support IRE ablation in small (<3 cm), non-resectable, primary or secondary liver cancers, although larger randomized trials are needed to determine the relative efficacy of IRE versus existing thermal-ablative methods.

## 3. Irreversible Electroporation in Whole-Organ Engineering

Organ transplants remain the definitive treatment for patients with end-stage organ dysfunction. The need for donor organs is increasing at a rapid rate with the demand for organs outpacing the available supply. This has prompted research into various organ alternatives including extracorporeal bioartificial liver devices [30], implantation of engineered tissues, and cell-based therapies. In recent years, the creation of a scaffold-based microenvironment that retains the three-dimensional structure and vasculature of a tissue or organ, while allowing for effective engraftment of cells, has become a key area of interest. Allogenic and xenogenic donor organs are being increasingly studied as potential sources of an organ derived ECM that could act as a biological scaffold. Upon decellularization of these organs through a variety of methods, including IRE, the resulting ECM scaffold can then be recellularized with engrafted cells. In addition to offering structural and mechanical support, this three-dimensional ECM “template” could also serve crucial biological signaling functions needed for, among other things, cellular differentiation and proliferation. Furthermore, decellularization of tissues and organs circumvents issues with immune rejection or cross-contamination in the case of xenogenic organ scaffolds, which has enabled it to become an emerging strategy in the creation of three-dimensional scaffolds and whole-organs. Here, we review a number of preclinical studies that examine the use of IRE in tissue decellularization. We will also briefly summarize tissue recellularization techniques, sources of cells, cell seeding, and perfusion culture. 

### 3.1. Decellularization

Decellularization is the process of removing all cellular components from a tissue or organ while retaining its three-dimensional native architecture, composition, and signaling cues. This architecture allows for cell attachments, differentiation, and vascularization. The critical retention of native vasculature allows for subsequent perfusion, oxygenation, and effective recellularization. Various methods and techniques have been described for tissue and organ decellularization including detergent-based, enzymatic, and mechanical methods [31]. Each technique has its advantages and disadvantages. An overarching disadvantage of many decellularization methods is the potential for damage to the ECM components and cell membrane, which significantly hinders recellularization efforts. Additionally, residual cellular material after decellularization has been shown to affect tissue remodeling and may limit the benefits of ECM scaffolds [32]. Thus, techniques like IRE are gaining momentum, serving as an alternative strategy to decellularize tissues and whole organs.

**Figure 3:**
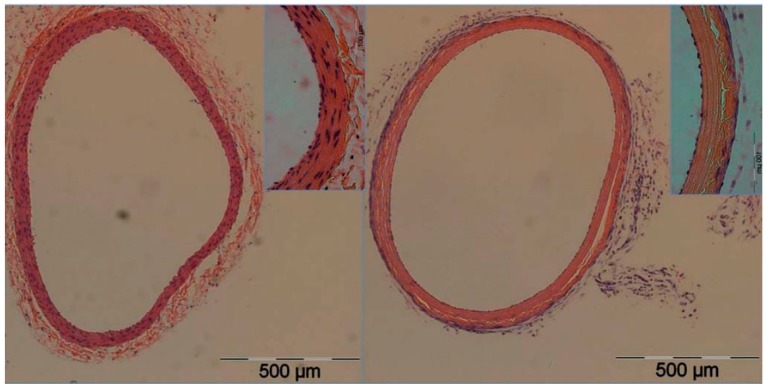
Total IRE-mediated ablation of vascular smooth muscle cells in rats. Cross-sectional image of rat carotid artery (right) after IRE-mediated total vascular smooth muscle ablation. Note the absence of vascular smooth muscle cells and repopulation of endothelial layer. The contralateral untreated carotid artery (left) was used as a control and demonstrates the abundance of vascular smooth muscle cells. Reproduced from with permission Maor, E. et al., *PLoS ONE*; published by Plos One, 2009.

One of the earlier studies to examine the role of IRE in organ decellularization was conducted by Maor et al. and examined the effect of IRE on rodent vascular smooth muscle cells [33]. In the common carotid arteries of Sprague–Dawley rats, IRE was performed under various field strengths and pulse rates followed by histological analysis of the ablated tissue. The results demonstrated complete ablation of the vascular smooth muscle cell population without damage to extracellular components (Figure 3). Moreover, the endothelial layer of cells exhibited complete regeneration after the IRE treatment, demonstrating the retention of the ECM’s functional capabilities after this cell-targeted ablative treatment [33]. In a similar analysis by the same group, Phillips and colleagues examined the efficacy of two different IRE methods in creating a decellularized arterial scaffold in vivo. In this experiment, IRE was delivered either through an electrode clamped to the exterior of a rodent carotid artery or through a minimally invasive endovascular approach in rabbit iliac arteries [34]. A subsequent histological analysis demonstrated significant decellularization of arteries treated with both approaches as well as endothelial regrowth seven days after treatment. Moreover, the ECM remained intact after treatment, showing no damage to the collagen and elastin fibers [34]. The investigators did note differences in the two IRE approaches, finding the external clamped electrode approach to have a more uniform electric field and less thermal tissue damage when compared with the endovascular approach. Nevertheless, both approaches demonstrated their efficacy in vivo, showing great promise in organ decellularization. 

In 2010, Sano et al. utilized a porcine liver model to study IRE decellularization. The investigators performed histological analysis on IRE treated centimeter-scale segments of porcine liver connected to a cardio-emulating perfusion system [35]. On histological examination 24 h post-treatment, they noted cell death in treated regions, but with a “preservation of major acinar features including connective tissue borders and blood vessels”, as well as biliary structures [35]. An analysis of the treated regions also demonstrated the maintained function of the vascular system in clearing cellular debris, although some remnant debris remained, raising the possibility of a subsequent immune response. Nevertheless, this analysis demonstrated the utility of IRE in whole segments of liver tissue. More recently, Zhang et al. performed a molecular and histological analysis on IRE-treated rat liver, seeking to elucidate the cell-level events that occur up to 24 h after ablation [16]. The investigators noted complete hepatocyte disintegration at 6 h post-treatment and by 24 h, new hepatocytes were seen in the treated region. These findings suggest that the optimal time for recellularization would be 24 h after IRE ablation [16]. Interestingly, Western blot analysis of the proteins involved in apoptosis, pyroptosis, and necroptosis revealed that pyroptosis and necroptosis were the primary forms of cell death in this study. They predominantly occurred up to 6 h after treatment. This finding is contrary to previous claims made in IRE literature claiming apoptosis as the primary form of cell death, and warrants further investigations into the exact mechanism of cell death in IRE.

Despite advances in IRE mediated decelluarization, the technique is not without limitations. The removal of cellular debris following IRE decellularization may be an immune mediated process, therefore, “the decellularization process would have to occur in vivo, thereby significantly limiting potential applications” [32]. Additionally, current IRE techniques utilize small probes and the efficient and effective decellularization of entire organs may be challenging. Nevertheless, IRE mediated decelluarization remains an active area of research with potential promise in regenerative medicine [36]. 

### 3.2. Recellularization

#### Sources of Cells

Upon decellularization with IRE, a three-dimensional ECM matrix can be recellularized with cells from a variety of sources. The ideal cell for recellularization would be one that can proliferate or self-renew as needed, and one that could give rise to the various types of cells needed to form a functional tissue or organ. Furthermore, the close interdependent relationship between extracellular support networks and functional parenchymal cells calls for careful review of donor recellularization agents. Stem cells that maintain indefinite proliferative and differentiable capability are ideal recellularization candidates [37]. Stem cells vary in origin and totipotency potential, offering the tissue engineer a vast repertoire of cells for consideration. Stem cells can be classified by the human host source (autologous or allogenic) and by age of acquisition (embryonic, fetal, or adult). Autologous cells are self-derived, consequently limiting immunogenicity, infection transmission, and the need for toxic immunosuppressive agents [38]. It is of particular note that eliminating the lifetime maintenance of antirejection therapy is enormously appealing to patients. The utility of autologous cells is limited by the difficulties associated with harvesting and obtaining sufficient quantities of cells. However, in the setting of the highly regenerative liver, autologous cells can be viably expanded for use in recellularization. Another possible option is allogeneic cells, which are derived from non-self and are thus genetically dissimilar. They can be harvested with greater ease and from healthy donors. They can also be isolated and expanded ex vivo for later use, such as in regenerative medicine. However, allogeneic cells can elicit life-threatening immune-mediated rejection reactions in patients and increase the risk of infectious agent transmission. 

Cell sources can be divided by embryologic, fetal, or adult origin. Embryonic stem cells are advantageous because they are pluripotent and relatively non-senescent [37]. Their use is complicated by ethical considerations and their immunogenic propensity (given their allogenic origin). Because they can differentiate into all three germ layers, they have a proclivity to form teratomas [37]. Fetal cells are multipotent and maintain proliferative capabilities, but historically have not been common targets for tissue engineering. They are often committed to a particular cell line and thus do not have a propensity to form teratomas and allow for easy harvesting. They have been used in applications such as bone and airway repair and hold promise for future clinical uses [39,40]. Inducible pluripotent stem cells, often of adult origin, have recently gained momentum as favorable contenders for tissue engineering. They can be induced into parenchymal or supportive cells. They also bypass the ethical concerns of embryonic and fetal stem cells. Additionally, their ability to grow in vitro allows for easy multiplication and the use of autologous, rather than allogeneic, cells. However, these cells often have epigenetic modifications that can potentially render them less safe and phenotypically less controllable than fetal or embryonic stem cells [41]. Adult-derived stem cells have been used clinically, particularly in the setting of bone marrow stem cell transplantation. They are easily harvested from adult donors, but their use is constrained by their narrow differentiable and proliferative profile. Other cells used in tissue engineering and therapeutic applications include umbilical cord blood cells and organ-derived progenitors. With all cell types, there remain important regulatory and economic issues to consider, including intellectual property rights, cost, and ethical concerns. 

## 4. Methods of Recellularization

Recellularization can be broken down into two phases: cell seeding and perfusion culture. Cell seeding is the repopulation of cells into a scaffold. The first important consideration in cell seeding is determining the required type and number of cells. Parenchymal cells, endothelial cells, and fibroblasts are all necessary to achieve operating status of engineered tissue. Parenchymal cells are necessary for functioning, endothelial cells ensure the organ matrix is non-thrombotic and protect parenchymal cells from shear stresses, and fibroblasts maintain the ECM. Cell number is another important parameter to ensure that the engineered organ can perform the desired biomechanical duties. For example, the liver of a human adult contains approximately 2.8 × 10^11^ hepatocytes [42]. Some studies indicate that 5% to 10% of the total liver weight is the minimum amount of tissue needed to improve the prognosis of a liver failure patient, which would amount to approximately 10^10^ cells. Seeding techniques in artificial organ design vary, but are mostly adaptations of methodologies currently used in cell-transplantation therapies. These techniques include intramural injection of cells or parenteral infusion of cells into blood vessels followed by continuous perfusion. Because whole-organ engineering is a field still in its infancy, ideal methods for recellularization are still unknown.

The second phase of recellularization is the perfusion culture. It is important to determine what culture conditions are necessary to facilitate cell uptake and growth. Bioreactors, such as the normothermic machine perfusion device (also known as organ culture), have historically been used extensively before the advent of cold storage devices to maintain ex vivo organ tissue. When configuring appropriate bioreactors, temperature conditions, perfusion of oxygen and nutrients, and elimination of waste buildup need to be considered. Centrifugal and positive-displacement pumps are two types of perfusion systems that have been employed to drive cells, nutrients, and oxygen through a tissue matrix. The composition of this perfusate is determined by media used in in vitro tissue culture for the respective cell type. Oxygen delivery is critical, and this parameter has been one of the main bottlenecks for development. 

## 5. Conclusions

IRE is a promising local ablative treatment for patients with liver tumors less than 3 cm and those adjacent to critical structures. Larger prospective studies stratified by primary tumor type, size, tumor biology, and previous or ongoing systemic treatments will help to elucidate the ideal patient population for IRE ablation. Randomized trials will also be needed to make direct comparisons with other local ablative methods. IRE has also demonstrated promise as a decellularization technique in whole-organ tissue engineering. By allowing for the formation of an acellular ECM undamaged by cell removal, IRE can facilitate research into whole-organ engineering and regenerative medicine. Further studies into ideal IRE techniques, mechanisms of recellularization, and cell sources are needed.
